# Knowledge and Awareness of the Association Between Periodontitis, Gastrointestinal Disorders, and Pancreatic Cancer: A Comparative Study Among Dental and Medical Postgraduate Students

**DOI:** 10.7759/cureus.113124

**Published:** 2026-07-21

**Authors:** Poorva Kulkarni, Sameer A Zope, Girish Suragimath, Siddhartha Varma, Apurva V Kale, Vaishali N Mashalkar

**Affiliations:** 1 Department of Periodontology, School of Dental Sciences, Krishna Vishwa Vidyapeeth (Deemed to be University), Karad, IND

**Keywords:** awareness, dental, gastrointestinal diseases, knowledge, medical, oral health, pancreatic cancer, periodontitis, students, systemic diseases

## Abstract

Background

Periodontal disease is increasingly recognized as a chronic inflammatory condition with potential systemic implications. Growing evidence suggests associations between periodontal disease and gastrointestinal (GI) disorders and pancreatic cancer, yet awareness of these links among future clinicians remains unclear.

Aim

This study aims to assess the awareness and knowledge regarding the correlation of periodontal disease with GI disorders, liver disease, and pancreatic cancer among dental and medical postgraduate students in Maharashtra.

Materials and methods

This cross-sectional, questionnaire-based study was conducted among 202 postgraduate students, including 108 dental and 94 medical postgraduates. A validated 18-close-ended questionnaire was distributed through WhatsApp. The questionnaire assessed demographic details, awareness, and knowledge regarding the relationship between periodontal disease and the selected systemic conditions. Data were analyzed using descriptive statistics and the chi-square test, with p < 0.05 considered statistically significant.

Results

Medical postgraduates showed significantly higher overall awareness of the systemic implications of periodontal disease than dental postgraduates (4.37 ± 3.09 vs 5.48 ± 2.41, p < 0.001). However, the overall knowledge scores between the two groups were comparable and did not differ significantly (3.79 ± 1.34 vs 4.23 ± 1.90, p = 0.058). Awareness of the association between periodontal disease and GI cancers was limited in both groups. Knowledge regarding the links between periodontal disease, liver disease, and pancreatic cancer was also variable, with several statistically significant differences observed between the two groups in disease-specific responses.

Conclusion

Medical postgraduates demonstrated greater awareness than dental postgraduates; however, knowledge regarding the association between periodontitis, GI disorders, and pancreatic cancer was moderate and inconsistent in both groups.

## Introduction

Periodontal disease (PD) is a chronic inflammatory condition characterized by the destruction of the supporting structures of the teeth, including the gingiva, periodontal ligament, cementum, and alveolar bone. Traditionally considered a localized oral infection, PD has now been increasingly recognized as a potential contributor to systemic health conditions through complex biological mechanisms involving microbial dysbiosis, immune-inflammatory responses, and their resultant systemic dissemination of pathogenic bacteria and proinflammatory mediators [1]. This evolving understanding places periodontal disease at the forefront of systemic disease research, emphasizing the bidirectional relationships between oral and systemic health [2].

Among systemic conditions linked with periodontal disease, gastrointestinal (GI) disorders, liver diseases, and pancreatic cancer have attracted significant scientific interest due to the shared inflammatory pathways, microbiome interactions, and/or exacerbating relationships [3]. The oral cavity serves as the anatomical and ecological gateway to the digestive system, allowing oral pathogens such as *Porphyromonas gingivalis*, *Fusobacterium nucleatum*, and other keystone species to translocate into the GI tract [4]. This can lead to alterations in the gut microbiome and mucosal immune dysregulation, which are implicated in the pathogenesis or progression of inflammatory bowel diseases (IBD) like Crohn’s disease and ulcerative colitis [5,6]. Furthermore, studies have indicated that chronic periodontal inflammation contributes to systemic endotoxemia, exacerbating hepatic inflammation and fibrosis, thus linking PD with conditions such as nonalcoholic fatty liver disease (NAFLD) and other chronic liver disorders [7,8].

Recent research has extended these associations to oncological implications, particularly the correlation between periodontal disease and pancreatic cancer. Emerging evidence suggests that oral microbiota and periodontal pathogens may contribute to carcinogenesis through mechanisms involving chronic systemic inflammation, microbial metabolites, and immune evasion [9,10]. These pathophysiological links highlight the importance of understanding the multifaceted role of periodontal disease in systemic disease etiology, prognosis, and therapeutic strategies [11].

Despite burgeoning evidence underscoring systemic ramifications of oral diseases, awareness and comprehension of these complex interrelationships among healthcare professionals vary considerably [12]. Postgraduate students in dental and medical disciplines, as future clinical and academic leaders, play a critical role in adopting a holistic approach to patient care integrating oral-systemic health. However, studies assessing the level of knowledge, attitudes, and awareness in this cohort concerning the correlation between periodontal disease and systemic conditions remain limited.

Evaluating the knowledge base of postgraduate students is essential to identify educational gaps and to promote interdisciplinary collaboration, clinical vigilance, and holistic healthcare delivery. Enhancing awareness about these interconnections can foster early detection and preventive strategies, reduce systemic disease burden, and improve overall patient outcomes.

This study aims to critically assess the extent of awareness and knowledge regarding the correlation of periodontal disease with GI disorders, liver diseases, and pancreatic cancer among dental and medical postgraduate students.

## Materials and methods

Study design

This cross-sectional, descriptive questionnaire-based study was conducted among dental and medical postgraduates from various colleges in Maharashtra. The questionnaire was disseminated through various online platforms to dental and medical postgraduate students across Maharashtra over a three-month period, from August 2025 to October 2025. A participant information sheet was provided, and informed consent was obtained from all participants before enrollment. The questionnaire consisted of three domains: demographic data, awareness, and knowledge about the correlation of the diseases. The first part included general demographic questions, such as the course, year, and college of the respondents. The second part focused on awareness, and the third part focused on knowledge about the correlation of periodontal disease with GI disorders, liver diseases, and pancreatic cancer. The inclusion criteria consisted of dental and medical postgraduate students across all years who voluntarily consented to participate. Interns, undergraduate students, and faculty members were excluded from the study. Additionally, individuals who were unwilling to participate or who failed to complete the questionnaire were excluded from the final analysis.

Sample size estimation

Using the single-proportion formula, for the sample size n at 95% confidence interval: $n = \frac{N \times X}{X + N - 1}$

$n = \frac{(Z_{\alpha/2})^2 \, P(1-P)}{d^2}$, where Zα/2 is the critical value of the normal distribution at α/2. The total sample size is calculated to be 194 (n = 194).

Ethical approval

The ethical clearance was obtained from the university ethics committee of Krishna Vishwa Vidyapeeth (Protocol no: 069/2025-2026) before commencing the study.

Prevalidation and pretesting of the questionnaire

A specially designed, closed-ended questionnaire (Google Forms, Google LLC, Mountain View, CA, USA) consisting of 18 questions was developed. It was pretested and validated by experts from the Krishna Vishwa Vidyapeeth protocol committee for content and face validity. The questionnaire was subjected to validation before data collection. Internal consistency reliability was assessed using Cronbach’s alpha, which yielded a value of 0.86, indicating good reliability. Test-retest reliability was evaluated by administering the questionnaire to 30 participants at a two-week interval, resulting in an intraclass correlation coefficient (ICC) of 0.88, demonstrating good temporal stability. Content validity was established through expert review by five subject experts, who assessed the relevance, clarity, and comprehensiveness of the questionnaire items. Necessary modifications were incorporated based on their recommendations. A pilot study was subsequently conducted on 30 participants to assess the clarity, comprehensibility, and feasibility of the instrument before its use in the main study.

Distribution of the questionnaire

The questionnaires were distributed through multiple online platforms to medical and dental postgraduate students from various colleges across Maharashtra. The study employed a nonprobability sampling approach. The questionnaire was disseminated electronically among dental and medical postgraduate students through online platforms using convenience sampling. Participants were further encouraged to share the questionnaire with their colleagues, thereby incorporating a snowball sampling strategy to enhance participant recruitment. Each participant received a message containing an electronic informed consent form and a secure link to the questionnaire. Only responses from individuals with completed both the consent form and the questionnaire were included in the statistical analysis. To minimize the risk of duplicate entries, the Google Form was configured to allow only one response per participant.

Scoring

The questionnaire consisted of 10 awareness-related items and eight knowledge-related items. Each correct response was assigned a score of 1, while each incorrect response was assigned a score of 0. Individual awareness and knowledge scores were calculated by summing the number of correct responses.

To facilitate interpretation of the findings, the total scores were converted into percentages. A score of 50% was considered the minimum acceptable level of awareness and knowledge. Accordingly, participants scoring ≥5 out of 10 on awareness items were categorized as having adequate awareness, whereas those scoring <5 were categorized as having inadequate awareness. Similarly, participants scoring ≥4 out of eight on knowledge items were categorized as having adequate knowledge, while those scoring <4 were categorized as having inadequate knowledge.

Statistical analysis

The collected responses were organized in MS Excel (Microsoft Corporation, Redmond, Washington, United States) and analyzed using IBM SPSS Statistics for Windows, Version 24 (Released 2016; IBM Corp., Armonk, New York, United States). Basic data were presented as percentages. The Pearson Chi-square test was used to compare groups based on knowledge and awareness. A p-value of less than 0.05 was considered statistically significant.

## Results

**Figure 1:**
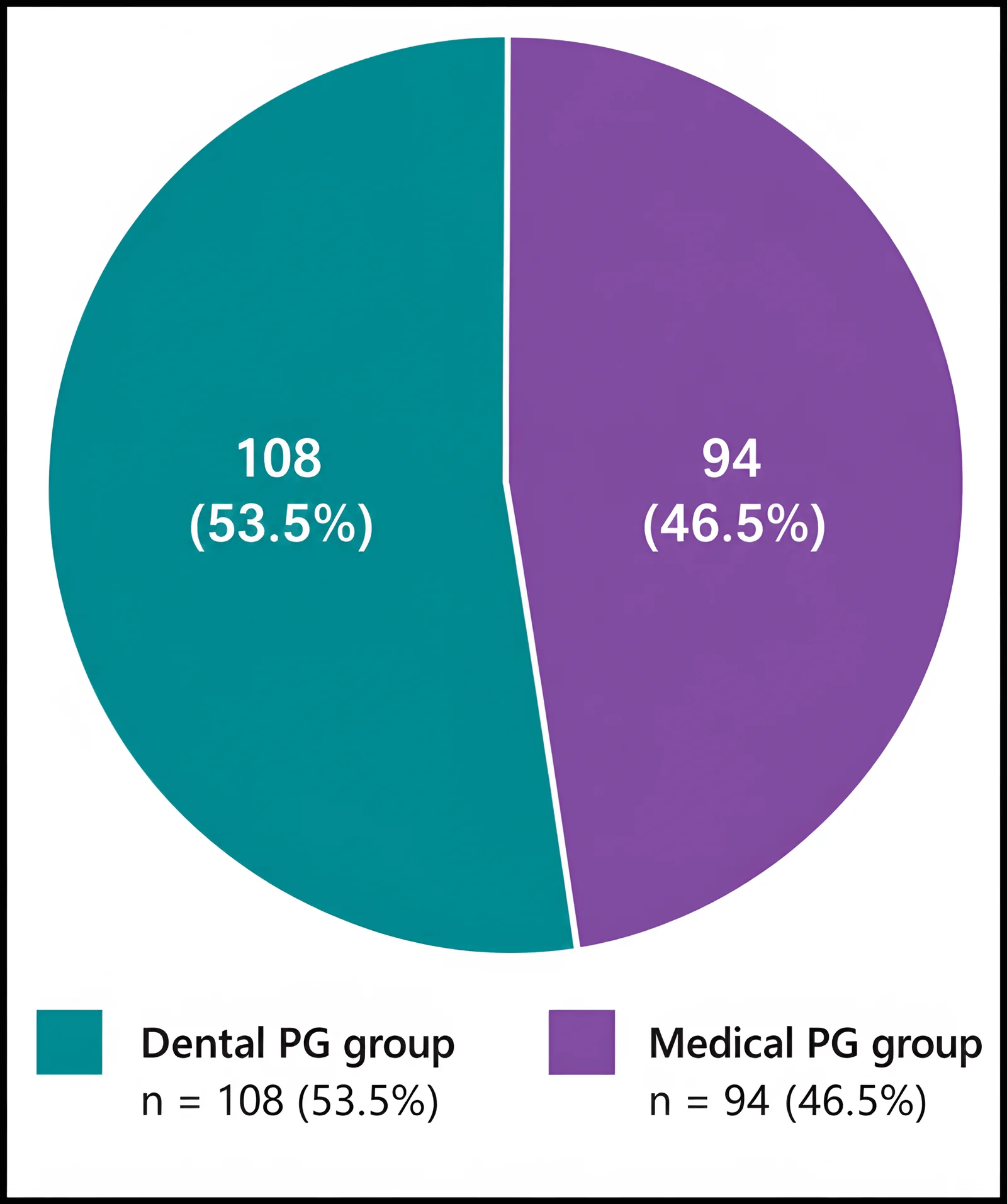
Distribution of postgraduates by specialty PG: postgraduate

A total of 202 (n = 202) postgraduate students participated in the study, including 108 (53.5%) dental postgraduates and 94 (46.5%) medical postgraduates (Figure 1). All the accepted questionnaires were complete and suitable for statistical analysis. 

Table 1 depicts the distribution of dental and medical postgraduate students based on the overall awareness and knowledge levels. Among the dental postgraduate students, 48.1% demonstrated adequate awareness, while 51.9% exhibited inadequate awareness. In contrast, a higher proportion of medical postgraduate students demonstrated adequate awareness (61.7%), with 38.3% categorized as having inadequate awareness.

**Table 1:** Distribution of participants according to overall awareness and knowledge levels PG: postgraduate

Domain	Group	Adequate n (%)	Inadequate n (%)
Awareness	Dental PG group	52 (48.1%)	56 (51.9%)
	Medical PG group	58 (61.7%)	36 (38.3%)
Knowledge	Dental PG group	58 (53.7%)	50 (46.3%)
	Medical PG group	61 (64.9%)	33 (35.1%)

With respect to knowledge, 53.7% of dental postgraduate students demonstrated adequate knowledge, whereas 46.3% had inadequate knowledge. Similarly, among medical postgraduate students, 64.9% demonstrated adequate knowledge, and 35.1% demonstrated inadequate knowledge. Overall, medical postgraduate students exhibited higher levels of both awareness and knowledge compared to dental postgraduate students regarding the association between periodontal disease and GI disorders. An independent t-test revealed a statistically significant difference (p < 0.05) in awareness of the systemic implications of periodontal disease between medical and dental postgraduate students (Table 2).

**Table 2:** Comparison of mean overall awareness scores between dental and medical postgraduate students PG: postgraduate; CI: confidence interval p < 0.05 was considered statistically significant; * indicates statistical significance

Group	Mean ± standard deviation	Standard error mean	Mean difference	95% CI		t value	p-value
				Lower bound	Upper bound		
Dental PG group	4.370 ± 3.098	0.298	-1.118	-1.897	-0.340	-2.83	0.005*
Medical PG group	5.489 ± 2.412	0.248					

When comparing the knowledge among the medical and dental postgraduates, the mean scores were comparable between both groups but statistically not significant (Table 3).

**Table 3:** Comparison of the mean overall knowledge scores between dental and medical post graduate students PG: postgraduate; NS: not significant p < 0.05 was considered statistically significant

Group	Mean ± standard deviation	Standard error mean	Mean difference	95% CI		t value	p-value
				Lower bound	Upper bound		
Dental PG group	3.796 ± 1.344	0.129	-0.437	-0.889	0.014	-1.909	0.058 NS
Medical PG group	4.234 ± 1.897	0.195					

Awareness and knowledge were evaluated across four domains that describe the association between periodontitis and systemic health, GI cancers, liver disease, and pancreatic cancer. Medical postgraduates demonstrated higher levels of awareness than dental postgraduates in the first three domains. Medical postgraduates more frequently recognized the link between periodontal disease and systemic health (77.7% vs. 45.4%, p < 0.001) and the association between poor oral hygiene and GI cancers (61.7% vs. 50.9%, p < 0.001). In the liver disease domain, medical postgraduates more often identified cirrhosis as a liver condition associated with poor periodontal health (60.6% vs. 37.0%, p < 0.004), acknowledged the role of the oral microbiome in liver disease (58.5% vs. 50.0%, p < 0.002), and recognized the bidirectional relationship between liver disease and periodontitis (48.9% vs. 40.7%, p < 0.001). However, within the pancreatic cancer domain, dental postgraduates showed comparatively greater awareness of the association between periodontitis and pancreatic cancer (37.0% vs. 27.7%, p < 0.001) and more frequently identified systemic disease as a shared risk factor (39.8% vs. 29.8%, p < 0.039) (Table 4).

**Table 4:** Responses of respondents in each question NS: not significant; PG: postgraduate Chi-square test (χ²); p < 0.05 was considered statistically significant; * indicates statistical significance

Question	Group	Option 1: n (%)	Option 2: n (%)	Option 3: n (%)	χ² value	p-value
AWARENESS						
Q1. Did you know that bacteria play a key role in causing periodontitis?	Dental PGs	Yes: 61 (56.5)	No: 28 (25.9)	Not sure: 19 (17.6)	21.489	<0.001**
	Medical PGs	Yes: 81 (86.2)	No: 9 (9.5)	Not sure: 4 (4.3)		
Q2. Are you aware of a possible link between periodontal disease and systemic health?	Dental PGs	Yes: 49 (45.4)	No: 26 (24.1)	Not sure: 33 (30.6)	46.162	<0.001**
	Medical PGs	Yes: 73 (77.7)	No: 17 (18.1)	Not sure: 4 (4.2)		
Q3. Are you aware of any association between periodontal diseases and gastrointestinal cancers?	Dental PGs	Yes: 54 (50.0)	No: 54 (50.0)	Not sure: 0 (0.0)	17.735	<0.001**
	Medical PGs	Yes: 44 (46.8)	No: 36 (38.3)	Not sure: 14 (14.9)		
Q4. Do you know that gastric ulcer has been most commonly associated with periodontitis in recent studies?	Dental PGs	Yes: 45 (41.7)	No: 63 (58.3)	Not sure: 0 (0.0)	17.906	<0.001*
	Medical PGs	Yes: 39 (41.5)	No: 42 (44.7)	Not sure: 13 (13.8)		
Q5. Can poor oral hygiene contribute toward gastrointestinal cancers?	Dental PGs	Yes: 55 (50.9)	No: 26 (24.1)	Not sure: 27 (25.0)	19.734	<0.001*
	Medical PGs	Yes: 58 (61.7)	No: 19 (20.2)	Not sure: 17 (18.1)		
Q6. Does oral microbiome have any role in liver disease development or progression?	Dental PGs	Yes: 54 (50.0)	No: 26 (24.1)	Not sure: 28 (25.9)	16.619	<0.002*
	Medical PGs	Yes: 55 (58.5)	No: 11 (11.7)	Not sure: 28 (29.8)		
Q7. Is the relationship between liver disease and periodontal disease bidirectional?	Dental PGs	Yes: 44 (40.7)	No: 0 (0.0)	Not sure: 64 (59.3)	17.375	<0.001*
	Medical PGs	Yes: 46 (48.9)	No: 11 (11.7)	Not sure: 37 (39.4)		
Q8. Is there any correlation between pancreatic cancer and periodontitis?	Dental PGs	Yes: 40 (37.0)	No: 32 (29.6)	Not sure: 36 (33.3)	22.376	<0.001*
	Medical PGs	Yes: 26 (27.7)	No: 29 (30.9)	Not sure: 39 (41.5)		
Q9. What could be the common risk factors between pancreatic cancer and periodontal disease?	Dental PGs	Microbial load: 37 (34.3)	Tobacco use: 28 (25.9)	Systemic disease: 43 (39.8)	8.367	<0.039*
	Medical PGs	Microbial load: 27 (28.7)	Tobacco use: 39 (41.5)	Systemic disease: 28 (29.8)		
Q10. What could be the potential mechanism of action for the development of pancreatic cancer and periodontal disease?	Dental PGs	Gut dysbiosis: 48 (44.4)	Chronic inflammation: 32 (29.6)	Not inter-related: 28 (25.9)	13.678	<0.003*
	Medical PGs	Gut dysbiosis: 39 (41.5)	Chronic inflammation: 46 (48.9)	Not inter-related: 9 (9.6)		
KNOWLEDGE						
Q11. Which bacterium is commonly found in both periodontal tissues and gastric lesions, particularly in individuals with gastric ulcer disease?	Dental PGs	E. coli: 28 (25.9)	H. pylori: 51 (47.2)	P. gingivalis: 29 (26.9)	12.339	<0.006*
	Medical PGs	E. coli: 12 (12.7)	H. pylori: 62 (66.0)	P. gingivalis: 20 (21.3)		
Q12. Which of the following systemic diseases has been suggested as being linked to both periodontitis and gastric disease?	Dental PGs	Diabetes mellitus: 57 (52.8)	Osteoporosis: 30 (27.8)	Hyperthyroidism: 21 (19.4)	11.293	<0.010*
	Medical PGs	Diabetes mellitus: 60 (63.8)	Osteoporosis: 10 (10.6)	Hyperthyroidism: 24 (25.5)		
Q13. Which of the following do you think could be the mechanism of action by which periodontal diseases may contribute to gastrointestinal cancers?	Dental PGs	Oral dysbiosis: 38 (35.2)	Bacterial translocation: 35 (32.4)	Not sure: 35 (32.4)	8.641	<0.034*
	Medical PGs	Oral dysbiosis: 48 (51.1)	Bacterial translocation: 27 (28.7)	Not sure: 19 (20.2)		
Q14. Which liver disease(s) do you think could be associated with poor periodontal health?	Dental PGs	Cirrhosis: 40 (37.0)	Hepatic cancer: 31 (28.7)	Not sure: 37 (34.3)	13.242	<0.004*
	Medical PGs	Cirrhosis: 57 (60.6)	Hepatic cancer: 20 (21.3)	Not sure: 17 (18.1)		
Q15. Which microorganism could be associated with nonalcoholic fatty liver disease and periodontal disease?	Dental PGs	A. actinomycetemcomitans: 46 (42.6)	E. coli: 27 (25.0)	P. gingivalis: 35 (32.4)	3.014	0.390 NS
	Medical PGs	A. actinomycetemcomitans: 38 (40.4)	E. coli: 21 (22.3)	P. gingivalis: 35 (37.2)		
Q16. What could be the possible mechanism of action by which periodontal disease may lead to liver disease?	Dental PGs	Bacteria in bloodstream: 29 (26.9)	Inflammatory mediators: 31 (28.7)	Both: 48 (44.4)	4.719	0.194 NS
	Medical PGs	Bacteria in bloodstream: 20 (21.3)	Inflammatory mediators: 21 (22.3)	Both: 53 (56.4)		
Q17. Which of the following is a shared risk factor for periodontal disease and liver disease?	Dental PGs	Alcohol use: 46 (42.6)	Age: 28 (25.9)	Sedentary lifestyle: 34 (31.4)	16.961	<0.001*
	Medical PGs	Alcohol use: 62 (66.0)	Age: 21 (22.3)	Sedentary lifestyle: 11 (11.7)		
Q18. In patients with advanced liver disease, which of the following oral manifestations is most commonly observed?	Dental PGs	Gingival hyperplasia: 53 (49.1)	Oral petechiae: 27 (25.0)	Candidiasis: 28 (25.9)	13.999	<0.003*
	Medical PGs	Gingival hyperplasia: 37 (39.4)	Oral petechiae: 43 (45.7)	Candidiasis: 14 (14.9)		

## Discussion

This cross‑sectional questionnaire‑based study evaluated awareness and knowledge of the correlation between periodontal disease and GI disorders, liver disease and pancreatic cancer among dental and medical postgraduate students in Maharashtra. Based on the available literature, no prior questionnaire‑based study appears to have concurrently evaluated medical and dental postgraduates across the four domains of periodontal links with systemic health, GI cancers, liver disease and pancreatic cancer.

Medical postgraduates demonstrated higher overall awareness of the systemic implications of periodontal disease than dental postgraduates. Nevertheless, both groups achieved only moderate knowledge scores for these associations, and many participants in both groups expressed uncertainty on the disease‑specific questions.

Bhardwaj et al. [12] reported that medical students were generally aware of a broad link between periodontal disease and systemic health but showed gaps in their understanding of specific systemic associations. In line with these findings, the present study found that medical postgraduates exhibited higher overall awareness than dental postgraduates, yet both groups demonstrated only moderate and often uncertain knowledge regarding concrete links with GI cancers, liver disease, and pancreatic cancer. 

In a study by Al Malak et al. [13], Lebanese university students showed a basic awareness of periodontal disease, but fewer than half recognized its relationship with systemic diseases; only a small fraction of participants identified a possible relationship with cancer. Dayakar et al. [14] likewise found that students from professional colleges in Dakshina Kannada, including medical and Ayurveda cohorts, had limited understanding of periodontal health and its systemic impact despite being future healthcare providers. More recently, Kattel et al. [15] showed that undergraduate medical students in Nepal accepted a general connection between periodontal and systemic health but had difficulty specifying concrete systemic outcomes, indicating that conceptual awareness often exceeds detailed, mechanism‑based understanding.

The “gum-gut axis” has been outlined by Byrd and Gulati [16], who describe a bidirectional link between periodontal inflammation, oral dysbiosis and GI disease, and later work has extended this to GI carcinogenesis, implicating periodontitis‑related dysbiosis in cancers of the esophagus, stomach, colon, pancreas, and liver via chronic inflammation, microbial translocation, and immune dysregulation. In the present study, only about half of postgraduate participants recognized any association between periodontal disease and GI cancers, and awareness of specific conditions such as gastric ulcer was even lower, indicating that these links are not yet widely appreciated at the postgraduate level. Baima et al. [17] summarized epidemiological and mechanistic data showing that periodontitis and dysbiotic oral microbiota, particularly* Porphyromonas gingivalis* and *Fusobacterium nucleatum*, can translocate to the GI tract, disrupt the gut microbiome and promote inflammatory bowel disease and several GI malignancies. The uncertain awareness of these links among postgraduate students indicates that such concepts are not yet completely embedded in routine postgraduate teaching.

A similar discrepancy between evidence and awareness emerged for liver disease. In this study, medical postgraduates more often linked cirrhosis and liver disease progression with poor periodontal health and a possible role of the oral microbiome, whereas dental postgraduates showed more variable and uncertain responses. However, works by Hatasa et al. [7] and Alazawi et al. [8] have demonstrated associations between periodontitis, NAFLD, and hepatic fibrosis, and experimental and clinical data increasingly support an oral-gut-liver axis through which periodontal pathogens and systemic inflammatory mediators can aggravate liver injury. Reviews of oral dysbiosis in liver cirrhosis also report higher prevalence and severity of periodontal disease in cirrhotic patients, suggesting a clinically meaningful bidirectional relationship. The limited recognition of this relationship in the present sample, therefore, reflects an educational lag relative to the current strength of the evidence.

Epidemiological evidence, including the meta‑analysis of Maisonneuve et al. [18], supports an increased risk of pancreatic cancer in individuals with periodontitis. However, only a minority of postgraduates in our study were aware of this association, and many remained unsure, indicating an educational gap between the research literature and current postgraduate understanding. Subsequent narrative syntheses and microbiome‑based studies have further suggested that poor oral health and specific periodontal pathogens may contribute to pancreatic carcinogenesis via chronic systemic inflammation, immune modulation and microbially mediated effects on the tumor microenvironment. The very limited recognition of any periodontal-pancreatic link in the present postgraduate cohort underscores how slowly such newer associations are incorporated into educational frameworks.

Taken together, this study and the available literature indicate that while postgraduate students increasingly acknowledge that periodontal disease is systemically relevant, their knowledge of specific associations with GI disorders, liver disease, and pancreatic cancer remains incomplete and uncertain. These findings highlight the need for structured, evidence‑based integration of oral-gut, oral-liver and oral-oncologic concepts into both dental and medical postgraduate curricula, ideally through interdisciplinary, case‑based teaching that explicitly links periodontal status with GI and hepatopancreatic diagnoses and management.

Limitations and future perspectives

This cross-sectional, self-administered online survey can only describe associations at a single time point and is vulnerable to selection and information bias. As the sample included postgraduate students from only one Indian state, the findings may not be generalizable to other regions or educational contexts. The study assessed only awareness and knowledge and did not evaluate attitudes, clinical practices, or competency related to the periodontal-systemic health association.

Future work could recruit multicenter samples from varied regions and specialties to strengthen generalizability and enable subgroup comparisons. Studies using longitudinal designs can test how targeted educational interventions change awareness, knowledge, and related decisions. Objective measures such as referral patterns, joint case discussions, and routine inclusion of oral-systemic links in care would help capture actual changes in practice.

## Conclusions

The study demonstrated that medical postgraduate students exhibited higher overall awareness of the systemic relevance of periodontal disease than dental postgraduate students. However, both groups demonstrated inadequate knowledge regarding their specific associations with GI disorders, liver disease, and pancreatic cancer. These findings highlight existing knowledge gaps related to the oral-gut axis, liver disease, and pancreatic cancer-associated periodontal links. The results underscore the need for greater emphasis on evidence-based oral-systemic health education within both medical and dental curricula and support the promotion of interdisciplinary learning to enhance understanding of these associations.
